# Clear cell renal carcinoma metastasis to the thyroid gland combined with papillary thyroid carcinoma - a case report

**DOI:** 10.3389/fonc.2025.1704830

**Published:** 2026-01-09

**Authors:** Jasna Mihailovic, Sladjana Novkovic-Ostojic, Ivana Starcevic, Jelena Roganovic, Maja Stankov, Tijana Vasiljevic, Mirjana Zivojinov

**Affiliations:** 1 Department of Nuclear Medicine, Faculty of Medicine, University of Novi Sad, Novi Sad, Serbia; 2 Division of Nuclear Medicine, Oncology Institute of Vojvodina, Sremska Kamenica, Serbia; 3 Radiology Centre, Institute for Pulmonary Diseases of Vojvodina, Sremska Kamenica, Serbia; 4 Radiology Centre, University Clinical Centre of Vojvodina, Novi Sad, Serbia; 5 Department of Pathology, Faculty of Medicine, University of Novi Sad, Novi Sad, Serbia; 6 Department of Pathology and Laboratory Diagnostic, Oncology Institute of Vojvodina, Sremska Kamenica, Serbia; 7 Center for Pathology and Histology, University Clinical Center of Vojvodina, Novi Sad, Serbia

**Keywords:** clear cell renal carcinoma, metastases, papillary thyroid carcinoma, thyroid gland, thyroidectomy, tyrosin kinase inhibitors

## Abstract

Clear cell renal cell carcinoma (ccRCC) represents the most common subtype of renal malignancy. Although nephrectomy remains the standard primary treatment, distant metastases may occur years after the initial diagnosis. Metastasis of ccRCC to the thyroid gland is an uncommon clinical event. We report a rare case involving concurrent metastatic ccRCC to the thyroid combined with a papillary thyroid carcinoma. A 61-year-old male with a prior history of metastatic ccRCC to the pancreas was referred to our institution for evaluation of thyroid enlargement. The patient underwent thyroid lobectomy, and histopathological analysis confirmed metastatic ccRCC within the thyroid tissue. Nine months postoperatively, routine ultrasound follow-up revealed a multinodular goiter in the contralateral thyroid lobe. Following completion thyroidectomy, histological examination identified a papillary thyroid carcinoma, staged as pT1a. Due to disease progression and recurrence of pancreatic metastasis, the patient was subsequently treated with tyrosine kinase inhibitor therapy. At the most recent follow-up, the patient remained alive and in good clinical condition. Given that ccRCC can metastasize many years after the initial diagnosis, prolonged follow-up is essential, particularly in patients presenting with thyroid nodules. Since metastatic lesions may mimic primary thyroid malignancies, accurate preoperative diagnosis, including fine-needle aspiration cytology and cell block with immunohistochemistry, is critical for guiding management. Total thyroidectomy remains the most effective treatment for isolated ccRCC metastasis to the thyroid gland.

## Introduction

Renal cell carcinoma (RCC) accounts for about 90% of all kidney cancers, while clear cell renal cell carcinoma (ccRCC) represents approximately 70% of RCC cases (1). Clear cell renal cell carcinoma typically affects older adults, with the median age at diagnosis around 62 years, and shows a greater incidence in males (2). At the time of diagnosis, about 30% of patients present with synchronous metastases. Approximately 25–41% of patients may develop metachronous metastases years after nephrectomy with curative intent (3). Clear cell renal cell carcinoma usually metastasizes to multiple sites, most frequently involving the lungs, lymph nodes, bones, liver, adrenal glands, and brain (4, 5). In contrast, isolated (solitary) metastases are rare, occurring in only 1–4% of cases (4), with the thyroid gland being affected in approximately 0.2–1% of patients (6, 7).

Diagnosing ccRCC metastasis to the thyroid gland remains a clinical challenge. This is primarily because such cases are often asymptomatic, while current imaging methods cannot reliably distinguish primary thyroid malignancies from metastatic lesions. The standard diagnostic approach typically includes neck ultrasonography (US), contrast-enhanced computed tomography (ceCT), magnetic resonance (MR), and, in selected cases, F-18 fluorodeoxyglucose positron emission tomography/computed tomography (F-18 FDG-PET/CT) scanning. However, these imaging techniques are non-diagnostic and cannot differentiate primary from secondary thyroid neoplasms. Ultrasonographic characteristics of thyroid metastases from various primary tumors are generally non-specific. They often present as heterogeneous, hypoechogenic, hyper vascular lesions with non-circumscribed margins and no calcifications (8–10). Reports from several authors describe thyroid metastases from ccRCC as either solitary solid nodules without calcifications (11) or hypoechogenic lesions with well-defined margins and increased intranodal vascularity (12).

Fine−needle aspiration cytology (FNAC) is routinely included in the preoperative diagnostic algorithm of thyroid nodules and demonstrates high diagnostic accuracy. Its sensitivity and specificity range from 65% to 98% and 72% and 100%, respectively (13–15). When the Bethesda System is applied, sensitivity and specificity improve to 92.8% and 94.2%, respectively, with a false-negative rate of around 5.8% (14). Most studies indicate that establishing a definitive preoperative diagnosis is challenging, often necessitating intraoperative frozen sections and postoperative pathological evaluation, which may require additional confirmation by immunohistochemical (IHC) analysis (16–18). Metastatic ccRCC involving the thyroid typically demonstrates positive immunoreactivity for RCC marker, CD10, PAX-2, PAX-8, vimentin and carbonyc anhydrase IX (CA IX) and negative staining for thyroglobulin (TG) and thyroid transcription factor-1 (TTF-1) (18–21). Given the superior diagnostic accuracy of histopathology, some authors recommend considering diagnostic surgery for thyroid nodules in patients with a history of malignancy when FNAC results are inconclusive (8, 22).

Although the metastasis of the ccRCC to the thyroid gland have been previously documented (11, 16, 23–33), metastatic ccRCC to the thyroid gland coexisted with a primary papillary thyroid carcinoma (PTC) is exceptionally rare (19, 34–36). In this article, we present a rare case of ccRCC metastasis to the thyroid gland combined with papillary thyroid carcinoma.

## Case report

**Figure 1:**
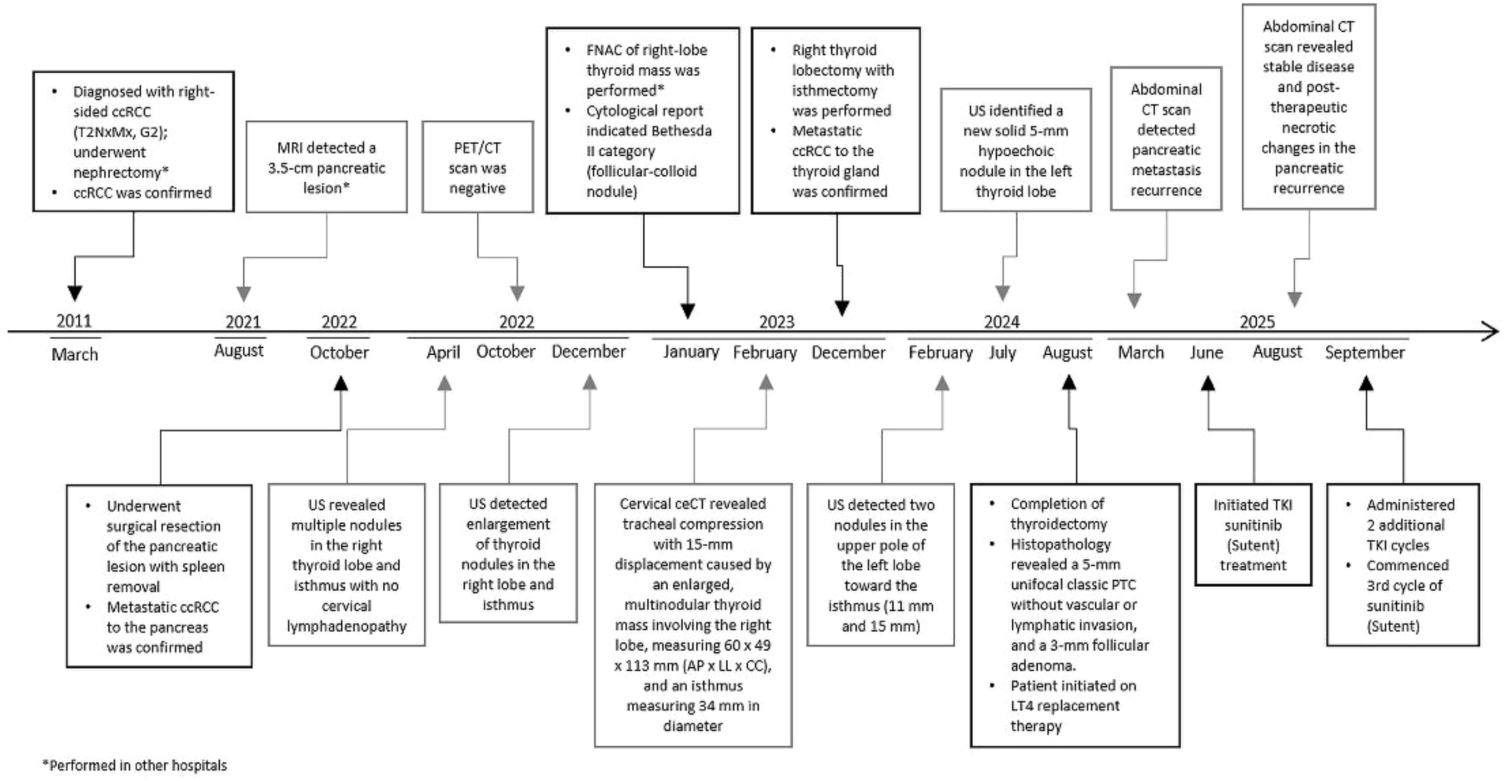
Patient medical history timeline.

We present the case of a 61-year-old male patient histologically diagnosed with clear cell carcinoma of the kidney following a right nephrectomy in 2011 in another institution, as T2NxMx, G2 (**Figure 1**).

Postoperatively, according to the institutional Tumor Board decision the patient did not receive additional treatment (chemotherapy and/or external beam irradiation) but was placed under regular surveillance by his referral physician.

**Figure 2:**
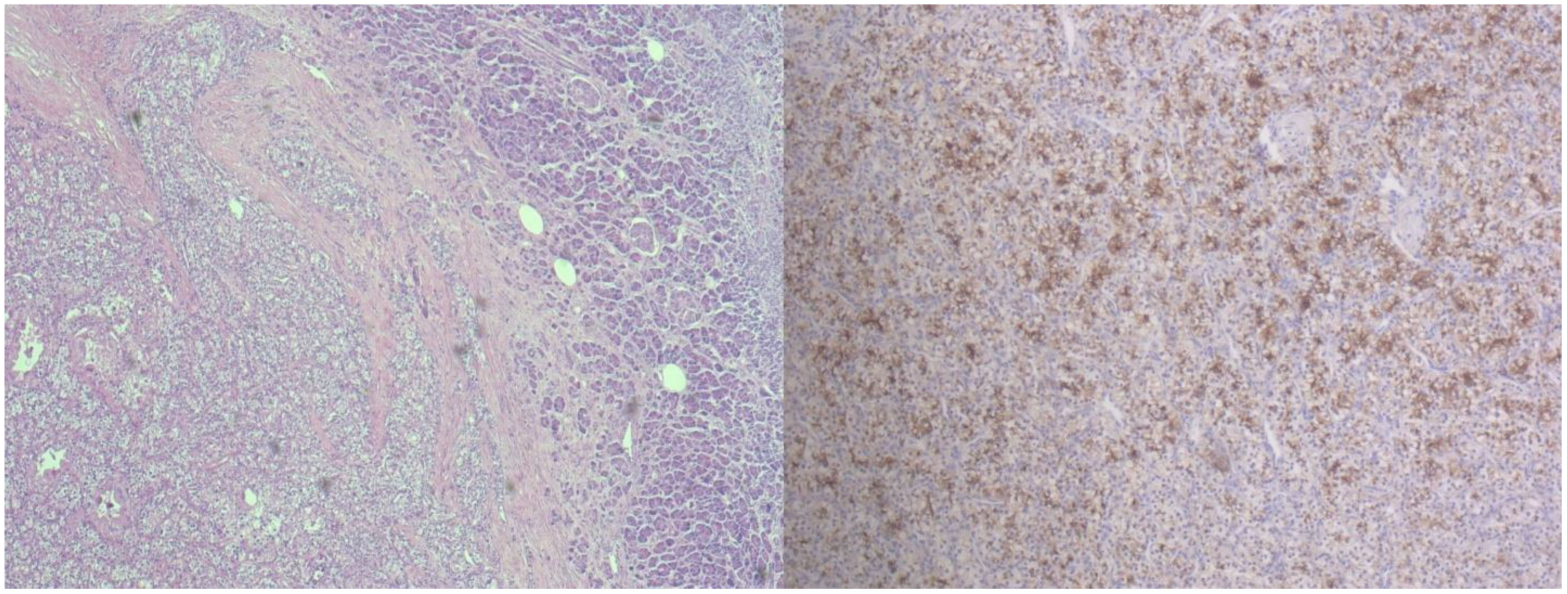
Metastatic RCC in pancreas. Left - There is an abrupt transition from pancreatic tissue to tumor made of polygonal tumor cells with extremely bright cytoplasm and hyperchromatic vesicular nuclei. The tumor cells are arranged in adenoid structures and solid plaques (5x, H&E). Right - Positive tumor cells in pancreatic metastasis stained for RCC (10x; IHC).

In 2021, an MR performed in another hospital detected a large pancreatic lesion with 3.5cm in size. Consequently, the patient underwent surgical resection of the body and tail of the pancreas. Histopathological analysis revealed multifocal metastatic clear cell carcinoma, confirmed with IHC (**Figure 2**).

In March 2022, the patient reported a sudden enlargement of the right side of the neck. Subsequent US, performed one month later, revealed multiple nodules in the right thyroid lobe and the isthmus with no cervical lymphadenopathy. Thyroid function tests showed normal thyroid hormones (thyroxine, triiod**o**thyronine) and thyroid**-**stimulating hormone levels and thyroid antibodies were negative. During routine follow-up, a control CT scan of the abdomen in June 2022 identified suspicious lymphadenopathy. In response, the radiation oncologist recommended F-18 FDG-PET/CT scan to evaluate the extent of the disease and guide treatment decisions. The PET/CT scan showed no FDG-avid foci suggestive of recurrent or metastatic disease, except for an enlarged right thyroid lobe and isthmus with mild metabolic activity (SUVmax ranging from 2.8 to 3.3).

**Figure 3:**
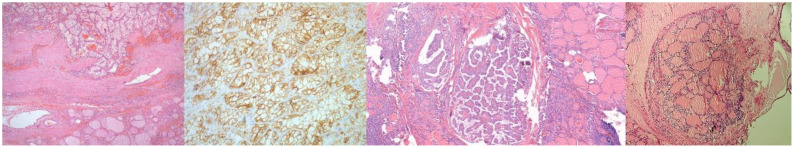
Thyroid with bilateral tumors. Left - Atrophic thyroid tissue with partially incapsulated tumor made of clear cells and vascular stroma in right lobe of the thyroid (10x; H&E). Middle left - Metastatic RCC in thyroid shows membrane positivity for RCC marker (20x; IHC). Middle right - Classic subtype of papillary thyroid carcinoma present in left lobe after totalization of thyroidectomy (10x, H&E). Right – Additional follicular adenoma 3 mm in diameter was found in the left lobe of the thyroid (10x; H&E).

However, a control US of the thyroid gland in December 2022 detected deterioration compared to findings from April 2022, suggesting progression of the thyroid mass involving right lobe and isthmus that warranted further investigation. In January 2023, at a different institution, US- guided FNAC of the thyroid mass in the right lobe was performed. The FNAC result was categorized based on the second edition of The Bethesda System for Reporting Thyroid Cytopathology, indicating Bethesda II category (follicular-colloid nodule) (37). A month later, in February 2023, a cervical ceCT revealed an enlarged multinodular thyroid mass involving the right lobe, measuring 60 x 49 x 113 mm (AP x LL x CC), and an isthmus measuring 34 mm in diameter. There was a compression and indentation of the tracheal wall, with the trachea displaced 15 mm contralaterally. The thyroid mass also displaced the common carotid artery (CCA) to the right and the sternocleidomastoid muscle (SCM) anteriorly. The patient subsequently, underwent a right thyroid lobectomy with isthmectomy at our institution in December 2023, and histopathological examination confirmed metastatic RCC in the thyroid gland. Metastatic node in the right lobe was solitary, noninvasive, circumscribed, and partially incapsulated tan node with larger areas of hemorrhage. Histological examination revealed a solid cell tumor composed of atypical cells with large clear cytoplasm and small pikenotic nuclei, prompting additional immunohistochemical analysis. Moderately differentiated metastatic clear cell renal cell carcinoma was immunohistochemically confirmed - tumor immunophenotype showed positivity for RCC and CD10, and negative for cytokeratin 7 (CK7), cytokeratin 20 (CK20), vimentin and TG (**Figure 3**).

**Figure 4:**
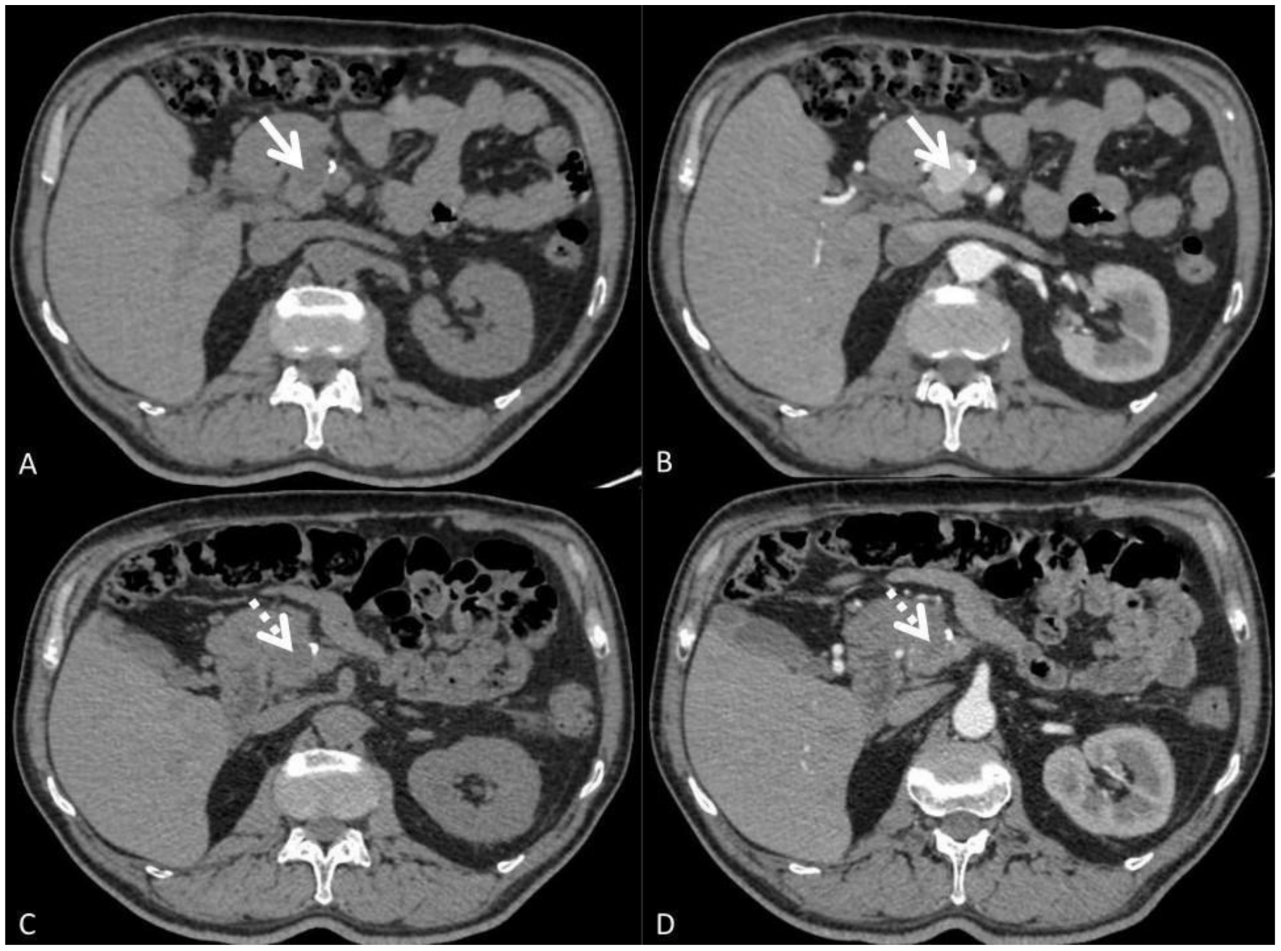
**(A–D)**. Axial non-contrast **(A)** and arterial phase **(B)** CT images of the abdomen reveal an avidly enhancing metastasis in the head of the pancreas measuring 2.5x 2.0 cm in diameter (arrow). Follow-up CT images **(C, D)** obtained after 2 months of treatment demonstrates decreased attenuation of metastatic lesion in the head of the pancreas with minimal size reduction to 2.2 x 1.7 cm (dashed arrow).

A follow-up US in February 2024 detected two confluent, well-demarcated nodules in the upper pole of the left thyroid lobe toward isthmic region, measuring 15 x 12 mm and 11 mm, with intranodular vascularization, while the left lobe appeared heterogeneous without focal lesions. A repeat US in February 2024 confirmed two confluent nodules in the upper pole of the left thyroid lobe toward isthmic region. On follow-up US in July 2024, two adjacent hypoechoic nodules measuring 11 x 7 mm and 15 x 11 mm were observed in the upper pole of the left lobe toward isthmic region, along with a newly developed 5-mm hypoechoic (cystic) nodule in the left lobe. Since the patient did not consent to FNAC and had a previous history of metastatic ccRCC to the thyroid, the nuclear medicine specialist recommended a further surgical consultation. As a result, a decision was made to surgically remove the remaining thyroid tissue. In August 2024, the patient underwent surgery. Definitive histopathological analysis of the resected left thyroid lobe revealed a 3-mm follicular adenoma and a 5-mm unifocal classic PTC confined to the thyroid without lymphatic or vascular invasion, classified as pT1a. Subsequently, he was started on levothyroxine (LT4) replacement therapy. During a routine follow-up in March of the current year, CT imaging revealed evidence of a recurrence of pancreatic metastasis. Based on the decision of the Institutional Tumor Board, the patient was referred for treatment with sunitinib, a tyrosine kinase inhibitor (TKI). After completing the second cycle of TKI therapy, a follow-up CT scan revealed stable disease and post-therapeutic necrotic changes in the secondary metastatic lesions located in the head and tail of the pancreas (**Figure 4**).

Given the patient’s good clinical condition, two additional cycles of TKI therapy were prescribed. At his most recent visit, the patient had commenced the third cycle of sunitinib (Sutent™, Pfizer). At present, patient is alive and in good condition. Close follow-up monitoring will continue in the future.

## Discussion

The incidence of metastases to the thyroid gland from non-thyroid tumors is uncommon in clinical practice. It is estimated to be 0.36% of all thyroid neoplasms, but may reach up to 1.4 - 25% in autopsy reports (38). Despite its rarity, ccRCC is the most frequent primary tumor to metastasize to the thyroid, accounting for approximately 48% of all thyroid metastases (8, 39).

The mechanism underlying metastatic spread to the thyroid gland remains unclear. Among some hypothesis, there is a theory suggesting that the rapid arterial blood flow, combined with high concentrations of oxygen, low concentrations of carbohydrate thyroid tissue and large amounts of iodine, makes the thyroid less susceptible to metastases (40). Additionally, it has been reported that renal cell carcinoma can metastasize to the thyroid by bypassing the lungs through the valveless paravertebral venous plexus of Batson (41).

Metastatic ccRCC to the thyroid gland often occurs several years after the initial diagnosis and treatment, varying from a short period of only 1–3 years (27–29), 5–16 years (16, 23– 25, 29, 31–33, 42–44), reaching up to 25 years (30). Metastatic ccRCC has been reported to metastasize into different thyroid lesions, including benign (follicular adenomas, Hürthle cell adenoma) and malignant tumors (PTC, follicular variant of PTC, oncocytic Hürthle cell carcinoma) (35). In this report, we present a case involving a 12-year latency period from nephrectomy to diagnosis of thyroid metastasis. Papillary thyroid carcinoma was identified 8 months after the diagnosis of the ccRCC-derived secondary thyroid neoplasm. Differentiating primary thyroid malignancies from metastatic thyroid lesions remains diagnostically challenging. FNAC is widely used to evaluate thyroid nodules and generally demonstrates high diagnostic accuracy. However, despite its usefulness in assessing thyroid tumors, FNAC has a limited ability to distinguish primary thyroid carcinoma from secondary metastases to the thyroid, largely because of its relatively high false-negative rate ranging from 1.5% to 50%. Chung et al. reported that preoperative FNAC yielded incorrect diagnoses in 26.3% of metastatic thyroid tumors, with RCC demonstrating the highest inaccuracy rate at 28.6% (8).In a study by Khaddour et al., metastatic cancer cells were found in only 29.4% of FNAC samples. FNAC missed nearly half (47.1%) of metastatic thyroid lesions, indicating limited sensitivity (45). Several authors have reported that the diagnostic utility of core needle biopsy is superior to that of FNAC. Song et al. found that FNAC results were 22% nondiagnostic, 44% benign, 22% atypia of undetermined significance, and 11% metastatic RCC. In contrast, all core-needle biopsies accurately confirmed metastatic RCC, even in cases missed by FNAC (12). Similarly, Choi et al. studied 46 patients with metastatic thyroid nodules from various primary origins. They demonstrated that core-needle biopsy had higher sensitivity than FNAC (100% *vs*. 58.6%, P = 0.008) and produced no false-negative results (46). Other studies and case reports have described even higher false-negative or indeterminate rates for metastatic RCC, reaching up to 82.3% (11, 46–51). Most inaccurate FNAC results are categorized as benign or indeterminate (11, 47, 49– 51). Several factors likely contribute to FNAC's low diagnostic yield in metastatic RCC including sampling errors, interpretive limitations, and the proportion of patients with benign FNAC results who proceed to surgical resection (13, 15, 52, 53). Moreover, ccRCC metastases to the thyroid are often highly vascular, leading to blood contamination and reduced cytologic clarity (54). In addition, FNAC provides only cytologic material, which is often insufficient for immunohistochemical staining (8, 22, 47).

According to current clinical guidelines, F-18 FDG PET/CT imaging is not routinely recommended for the initial assessment of ccRCC (55, 56). Available data on PET/CT use in metastatic ccRCC are limited, in part due to institutional follow-up protocols and the relatively low incidence of this malignancy. Although the diagnostic utility of FDG PET/CT in primary RCC is limited, its role in postoperative surveillance and in the early detection of ccRCC recurrence and metastases has been well established (57–62). Several studies have reported high sensitivity (74–100%) and specificity (80–100%) in detecting recurrent and/or metastatic disease (59–62). In addition, Pereira et al. reported that PET/CT is superior to ceCT for detecting both recurrent and metastatic lesions in RCC, with significantly improved specificity and negative predictive value (p < 0.0001) (63). According to some authors, FDG-avid thyroid nodules detected on PET/CT are highly suspicious of metastatic ccRCC to the thyroid and warrant subsequent FNAC (19, 64). However, metastatic thyroid lesions may be challenging to identify on imaging modalities such as PET/CT, as they can exhibit nonspecific metabolic activity that mimics benign lesions (65). In patients with a diagnosis of extrathyroidal malignancy, the presence of a thyroid mass or evidence of diffuse thyroidal uptake on F-18 PET/CT should raise awareness of metastatic involvement of the thyroid gland (66).

Although the benefit of total thyroidectomy compared with the subtotal thyroidectomy remains a matter of debate, most of existing literature supports surgical resection as the preferred intervention, primarily to mitigate the risk of developing aggressive locoregional disease (7, 17, 19, 23, 25, 26, 31, 33, 38, 67, 68). Thyroid metastases from ccRCC generally exhibit indolent clinical course and are associated with improved overall survival. This observation may be partially explained by underlying similarities in the molecular or genetic profiles of pancreatic and thyroid metastases in ccRCC (42, 67, 69, 70). Surgical resection of thyroid metastasis from RCC has been reported to confer a survival benefit, with a 4-year and 5-year survival rates of 53% and 30–60%, respectively (67, 68, 71–75). The postoperative radiotherapy is not recommended since RCC is resistant to radiation (26), while following current guidelines radioiodine therapy is not suggested in low-risk patients (76, 77).

In approximately 27% of patients diagnosed with RCC who develop thyroid metastases, additional metastatic sites have been reported, including the lungs, pancreas, skeletal system, lymph nodes, liver, and adrenal glands (71). Pancreatic metastases (PM) are rare, accounting for only 2–5% of pancreatic malignancies, with RCC being the most frequent cause, responsible for about half of all cases (78, 79). Pancreatic metastases from RCC can occur many years after nephrectomy- often more than 10 years and in some cases up to 33 years - and are usually metachronous (80). Although multiple metastases occur in 38% of cases, survival does not differ between single and multiple lesions (78). Pancreatic metastases from RCC often display indolent behavior and have a favorable outcome compared with metastases to other sites. As a result, PM in RCC is generally associated with a good prognosis (79, 81). Because PM-RCC may develop long after the primary tumor, they can be mistaken for primary pancreatic tumors without careful review of medical history. According to the WHO classification, the differential diagnosis of clear cell carcinomas in the pancreas includes: ductal adenocarcinoma with clear cell features, the solid variant of serous cystadenoma, clear cell solid pseudopapillary neoplasm, PEComa, clear cell pancreatic neuroendocrine tumor, and metastatic clear cell tumors (82).

Pancreatic metastases are typically highly responsive to surgical therapy, leading to long- term survival, in contrast to the aggressive biology and chemoresistance seen in primary pancreatic cancer. Surgical resection is usually the first line treatment and is associated with favorable outcomes, with reported 3- and 5-year overall survival rates of 72% and 63%, respectively (83, 84). According to the literature, the current first-line treatment for metastatic ccRCC is sunitinib malate, an oral multitargeted tyrosine kinase inhibitor with antitumor and anti-angiogenic activity that targets VEGFR, KIT, and PDGFR. In addition to angiogenesis inhibitors, rapamycin (mTOR) targeted inhibitors, and immune checkpoint inhibitors have been also approved by the Food and Drug Administration for the first-line treatment of patients with advanced ccRCC (85, 86). Previous literature data reported a 31% response rate with a median PFS of 11 months after the treatment with TKI (87).

## Conclusion

Metastatic clear cell renal cell carcinoma to the thyroid gland combined with papillary thyroid carcinoma is a rare occurrence, often presenting without specific clinical symptoms. In these patients, the priority is to determine the correct diagnosis and to differentiate metastatic ccRCC from primary clear cell neoplasm arising in the thyroid and pancreas. Although rare, pancreatic metastases can develop many years after the initial diagnosis of ccRCC and should be considered in any patient with a history of cancer who presents with an isolated pancreatic mass. Preoperative evaluation of the thyroid should include FNAC of any suspicious lesions, ideally combined with a cell block and IHC, to accurately differentiate primary from secondary thyroid malignancies. The final diagnosis of metastatic ccRCC should be made histologically following thyroid surgery. Due to the challenges in the preoperative diagnostic setting, total thyroidectomy seems to be the most appropriate surgical procedure. Ongoing long-term follow-up, along with diagnostic imaging and clinical assessment, is essential for optimal patient management and treatment outcomes.

## Data Availability

The raw data supporting the conclusions of this article will be made available by the authors, without undue reservation.
